# Poor welfare compromises testicle physiology in breeding boars

**DOI:** 10.1371/journal.pone.0268944

**Published:** 2022-05-26

**Authors:** Thiago Bernardino, Carla Patricia Teodoro Carvalho, Leonardo Batissaco, Eneiva Carla Carvalho Celeghini, Adroaldo José Zanella

**Affiliations:** 1 Department of Preventive Veterinary Medicine and Animal Health, School of Veterinary Medicine and Animal Science, University of São Paulo, Pirassununga, SP, Brazil; 2 Programa de Mestrado em Medicina Veterinária e Doutorado em Saúde Única, Universidade de Santo Amaro, São Paulo, SP, Brazil; 3 Laboratory of Semen Biotechnology and Andrology–Center of Biotechnology in Animal Reproduction, Department of Animal Reproduction, School of Veterinary Medicine and Animal Science, University of São Paulo, Pirassununga, SP, Brazil; 4 Laboratory of Teaching and Research in Pathology of Reproduction, Department of Animal Reproduction, School of Veterinary Medicine and Animal Science, University of São Paulo, Pirassununga, SP, Brazil; Humboldt-Universitat zu Berlin, GERMANY

## Abstract

In commercial pig breeding farms, boars are often exposed to stressful situations, such as confined housing conditions, inadequate environmental temperature, food restriction, lameness, diseases, among other challenges. Confined housing conditions, such as crates, are reported as a major source of stress for pregnant sows, and were banned in the UK and in Europe, however there is limited information about the impact of this housing system for boars. The goal of this study was to investigate the impact of three different housing conditions for boars and the consequence on the testicles. We studied 27 crossbred boars (F1 large white and landrace), housed in crates (n = 9), pens (n = 9), or enriched pens (n = 9), during 10 weeks. We collected data of scrotal superficies mean temperature (SSMT) with a thermal camera; we measured testicular parenchyma perfusion (ultrasound evaluation); and we measured sperm characteristics. We found that boars housed in crates had a higher SSMT (p < 0.05) and higher testicular parenchyma perfusion than boars housed in pens and enriched pens (p = 0.01). Regarding the semen features, we found that boars housed in crates showed more agglutinated semen, and higher values of linear curved linear velocity (VCL) than boars housed in pens and enriched pens, both indicators of reduced fertility. These results indicates that boars housed in pens and in enriched pens showed better indicators of testicular health, better sperm motility features (VCL, p = 0.046), and less agglutinated sperm (p < 0;05) than that observed in boars kept in crates.

## Introduction

In commercial pig breeding farms, boars are often exposed to stressful situations, such as confined housing conditions, inadequate environmental temperature, food restriction, lameness, diseases, among other challenges. The consequences of these welfare challenges are largely unknown and, eventually, could affect semen quality. Previous work demonstrated that housing boars in crates is stressful [1]. Boars housed in crates can experience reduced opportunities for testicular thermoregulation, due to limited locomotion, stress and limited physical space. There is much scientific evidence demonstrating that a poor thermal environment could compromise testis physiology and semen quality [1–3]. Moreover, it is already known that the paternal environment can influence the offspring behavior and metabolism. The most likely mechanism of this inheritance involve epigenetic, such as DNA methylation, chromatin remodeling, histone modifications, and regulation of gene expression by small non-coding RNAs [4].

In pregnant sows, the impact of housing conditions in their welfare has been extensively reported [5]. Housing pregnant sows in crates was prohibited in 2013 in the European Union, (EU Directive 2008/120 EC), as well as in some states in the USA. There is recent legislation in Brazil (IN113) addressing housing systems for sows, as well as for boars [6]. In the recently published IN113 [5], adult boars cannot be housed in pens smaller than 6 m^2^; this will come into force from the year 2045. The concern about the welfare of breeding boars is extremely limited over the globe. One of the reasons could be that the number of boars in pig farms is extremely small, when compared with the number of sows, piglets, weaners and fatteners. This is the case because there are pig farms that use boars only to detect estrous and this could partially explain why there are so few research groups investigating boar welfare. In addition, there is limited information about the impact of housing conditions on the health and and welfare indicators in boars. On the other hand, the number of descendants that each boar can generate in a year is much higher than for an individual sow, since artificial insemination increases the reproduction efficiency of males. If one boar mates three sows a week, in natural mating, it could father over 2,100 pigs in a year. If the semen is used in artificial insemination, depending on the technique (intracervical, post cervical or intrauterine), a single ejaculate can produce 20–60 inseminating doses [7]. This number represents a 20-fold increase of the production of a boar used in natural mating. Artificial insemination is one of the most efficient breeding methods for modern pig production [7].

Any factor that affects testis health can compromise semen quality in boars. The modern pig production industries, according to their technology status, demands high quality sperm and high fertility rates. Several protocols used to evaluate testicle condition involve manipulation, invasive techniques or uncomfortable handling of the animals. Thermography and ultrasonography are tools used to evaluate testicular health. Thermography has been used for pigs to detect skin surface temperature, heat stress, and to diagnosis joint inflammation [8]. However, we did not find studies using thermography to assess health indicators in the boar testis.

Another highly sensitive, easily available and non-invasive tool to measure blood flow is the use of Doppler ultrasonography [9–11], which can measure testicular parenchyma, pampiniform plexus vascularization and the resistance index of the pampiniform plexus arteries [12, 13].

In this study, we hypothesized that the housing conditions can have an effect on the testis health and sperm production. We expect that boars which experience poor welfare, in this particular manuscript poor housing conditions, will present worse indicators of testicular health, sperm morphology, and sperm motility. Our aim was to investigate the reproductive impact of housing young boars in crates, pens and enriched pens.

## Materials and methods

This study was approved by the Committee on Ethics in Animal Use (CEUA) of the School of Veterinary Medicine and Animal Science (FMVZ-USP), University of São Paulo, under protocol no. 3612010616.

### Experimental design

Boars were housed in three housing treatments: Crates: 9 animals housed in gestation crates, measuring 197 x 76 cm, half solid floor and half slatted flor (CR); pens: 9 animals housed in pens, measuring 241 x 376 cm (PE); and enriched pens: 9 animals housed in enriched pens, measuring 241 x 376 cm (EP). The pens had only solid floor with wired fences. For the enriched pens, environmental enrichment was offered twice daily, one hour after feeding represented by brushing the animals for two minutes using a broom, showering the animals with water for 30 seconds, and 500 grams of hay, approximately, was provided in the pens floor as rooting material.

**Fig 1:**
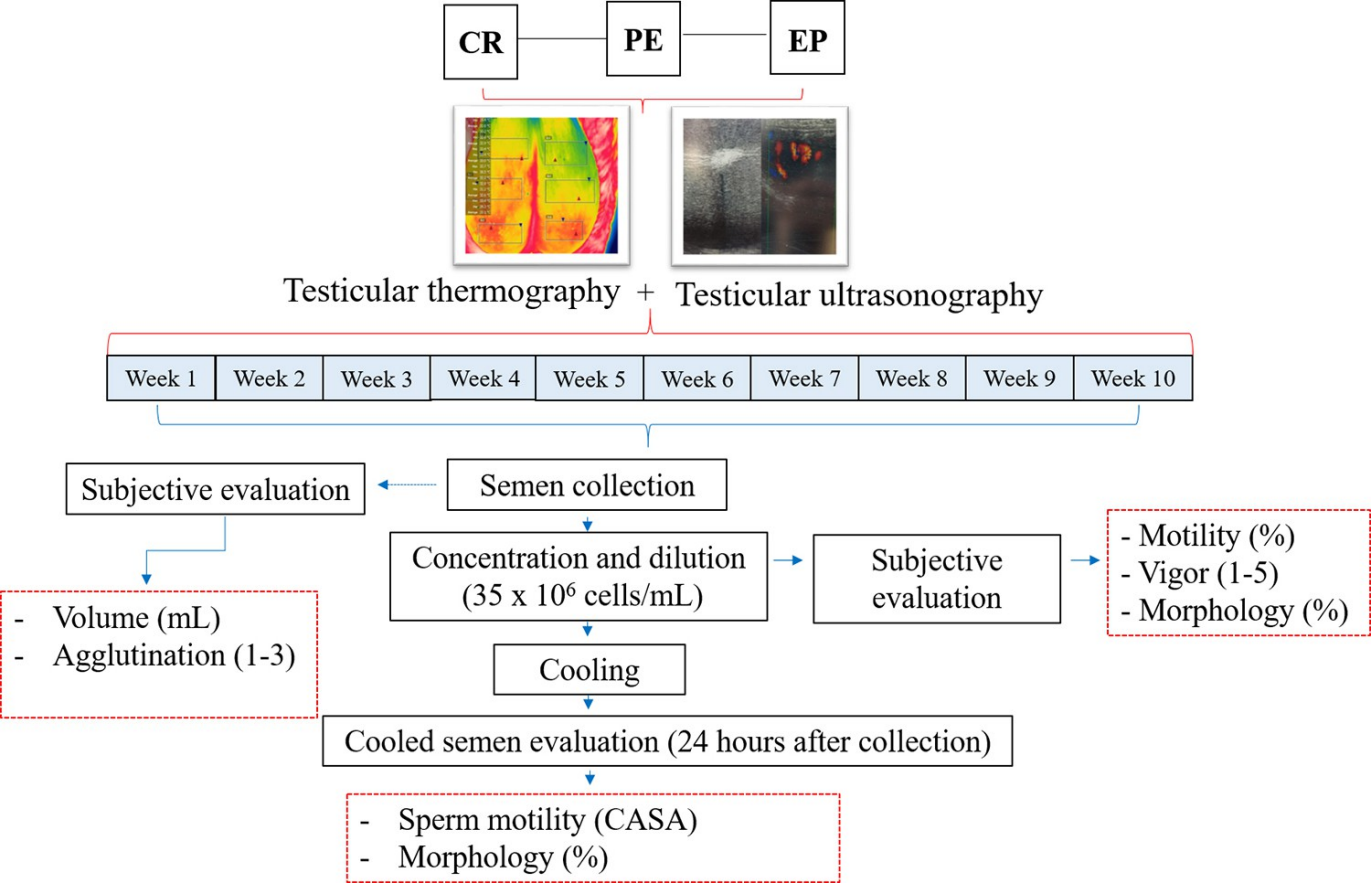
A representative diagram of experimental designs, sampling routine, and evaluated parameters.

The experimental design is shown in Fig 1. Our study compared the effect of the three housing treatments described over the time on the spermatic characteristics of the diluted semen and after cooling at 17°C, as well as the vascularization of testicular parenchyma, pampiniform plexus, and scrotal superficies mean temperature (SSMT). The evaluation of the vascularization of the testicular parenchyma, pampiniform plexus, and testicular temperature (SSMT) were carried out for 10 weeks, as well as the semen collection.

### Animals

For this study 27 hybrid boars (F1 large white x landrace) with an average age of 10 months were initially housed individually in pens, measuring 3.85 meters x 1.2 meters. Animals were fed twice daily, 07:00am and 01:00pm, with 2.8kg of concentrate per day, and had *ad libitum* access to water through a nipple drinker. All animals were kept in pens until the end of semen collection conditioning. Afterwards, animals were allocated to one of the three housing conditions. After the experimental period, all boars were euthanized by eletronarcosis (2.5 mAh), followed by a penetrative captive bolt (Accles & Shelvoke, Dispatch Kit. 25), and exsanguination.

### Treatments allocation

The allocation of the boars was based in their seminal quality and they were homogeneously distributed across the three treatments. The methodology used was adapted from Alves et al. [14] and Garcia (2004), based on a formula taking into account the total major defects, total minor defects, and progressive motility of sperm.

Boarscore=(1xPROG)+(3xMAJ)+(2xMIN)

where PROG was progressive motility, MAJ was major defects, and MIN was minor defects.

This approach was chosen in order to guarantee the presence of animals with good, medium and low semen quality in all groups. After the establishment of the semen quality boars with each classification were randomly assigned to one of the three treatment groups.

During the 10 weeks after treatment distribution, testicular (scrotal superficies mean temperature, SSMT) and body (eye area mean temperature, EAMT) temperature were measured weekly using thermography (T620 Flir Systems). In addition, bi-weekly assessment of the testicular hemodynamic characteristics using Doppler ultrasound (Mindray, model M5Vet, Digital Diagnostic Imaging System; 6LE5Vs Vet probe model) of the testicular parenchyma and pampiniform plexus were made. An initial measure was taken before treatment allocation.

### Semen sampling

Prior to the experimental study, six semen collections, with an average interval of 7 days between them, were carried out on all animals. Semen collection, semen analyses, and testicular thermography were performed weekly for 63 days, totaling an estimated 1.5 spermatogenesis and 6 epididymal transits. Thus, we analyzed 10 ejaculates for each of the 27 boars (n = 270), obtained by the gloved-handed method. Prior to moving the animal to the semen collection room, the boar was placed in a specific crate, had the preputial area cleaned with dry paper and the hairs on the prepuce were trimmed if necessary. We used a static mannequin, and the ejaculate was collected in a thermic recipient vessel (Equittec ®, Marau, Brazil), previously lined with a non-spermicide plastic collector, and a disposable filter (Equittec ®, Marau, Brazil) to separate the gelatinous fraction. Afterwards, the semen was taken to the laboratory attached to the collection room, and the volume was measures in a graduated beaker before taking samples for further analyzes.

### Semen analyses

The first analysis was regarded the agglutination. This feature was classified according the methodology described by Martín [15], and consisted of a score from 0 to 3, were 0 was no agglutinated cells and 3 represented more than 25% of agglutinated sperm cells. Afterwards, we measured the volume and semen aspect was observed (appearance, color, smell). Next, the sperm concentration (sperm per mililiter) and volume was evaluated. Sperm concentration (10^6^/mL) was determined after a dilution in 1:100 of semen in formaldehyde phosphate buffered saline (DPBS, Biodux^®^, Brazil; formaldehyde 4%). The counting was performed in a hemocitometric chamber (Neubauer chamber, HBG 9030–05, Germany) with a 22x22 millimeter coverslip and the number of cells per quadrant (5 on each side of the chamber) were measured and the concentration determined in optical microscopy (400x magnification).

Immediately after collection, the semen was filtered to separate the sperm rich fraction from the gelatinous fraction. After, the sperm rich fraction was diluted with specific diluent (Androstar ® plus, Minitube, Germany), the concentration was adjusted to 35 x 10^6^ sperm cells/mL. The next step was to analyze subjective sperm features, motility (%), vigor (1–5) and morphology (%). In order to motility and vigor analyses, aliquots were placed between the pre-heated slide and coverslip to assess under phase microscopy (100x magnification).

Sperm morphology was evaluated to guarantee that all samples showed a satisfactory pattern. The samples were diluted in pre-heated (37°C) formaldehyde phosphate buffered saline (DPBS, Biodux^®^, Brazil; formaldehyde 4%), and evaluated under differential interference contrast microscopy–DIC (Nikkon, Model Eclipse 80i, Tokyo, Japan) with 1,000x magnification, counting 200 cells per sample and classified in major, minor and total defects [16].

The semen was analyzed fresh, immediately after collection, and aliquots were frozen for epigenetic analyses. The samples were randomly collected within the treatments and the collection time, thus minimizing the effect of the time on the evaluated parameters. After preliminary analyses, the diluted semen samples were kept at 15°C, in a transport container BotuFlex^®^ (Botupharma, Botucatu-SP) for 24 hours, until the analyses. For the evaluation, an aliquot of 1 mL was kept in dry bath at 37°C during 90 seconds.

### Computer assisted sperm analyses

For the evaluation of motility, the computerized-assisted sperm analysis (CASA, HTM-IVOS-Ultimate Hamilton Thorne Biosciences, Beverly, MA, USA) with the setup previously adjusted for porcine semen analysis. The analyses were carried out at the Semen Biotechnology and Andrology Laboratory (LBSA), in the Department of Animal Reproduction (VRA) of the School of Veterinary Medicine and Animal Science (FMVZ) from the University of São Paulo (USP). For this evaluation, Leja^®^ slides chamber (IMV-Technologies International Corp.) was used at 37°C, placing 20μL of diluted semen preheated (37°C) in a dry water bath, and inserted in the IVOS equipment (Version 12.3, Hamilton-Thorne Bioscience^®^, Beverly, USA). This equipment analyzes the sperm by CASA, which captures images from the sperm cells through a microscope attached to the computer and transfers the data for analysis of the sperm movements through the *Animal motility* program, which was previous adjusted for porcine sperm analysis.

The CASA collected data and analyzed it from at least 5 different microscope fields. The following characteristics were analyzed: total motility (MT,%), progressive motility (PM, %), average path velocity (VAP, μm/s), straight line velocity (VSL, μm/s), curvilinear velocity (VCL, μm/s), amplitude of lateral head displacement (ALH, μm), beat-cross frequency (BCF, Hz), straightness of the average path (STR, %), linearity of the curvilinear path (LIN, %) and percentage of rapid cells (RAP, %). All the CASA analyses were performed 24 hours after dilution and cooling.

### Testicular thermography

Thermal pictures were collected using a T620 thermography camera (Flir Systems, USA). The distance between the animal and the camera was standardized at 1 meter and the camera emissivity was adjusted to 0.98. Prior to the picture collection, each animal was stimulated to stand up, had their scrotal area cleaned with a paper towel (when necessary) and animals were maintained standing for at least 10 minutes. The scrotal skin was not touched for at least 1 hour prior to image collection. Moreover, in order to keep the animals standing still, a small amount of their regular food was provided for each thermal image sampling. This approach allowed us to collect good quality pictures, inside their home pens, avoiding any kind of physical or pharmacological restraint.

**Fig 2:**
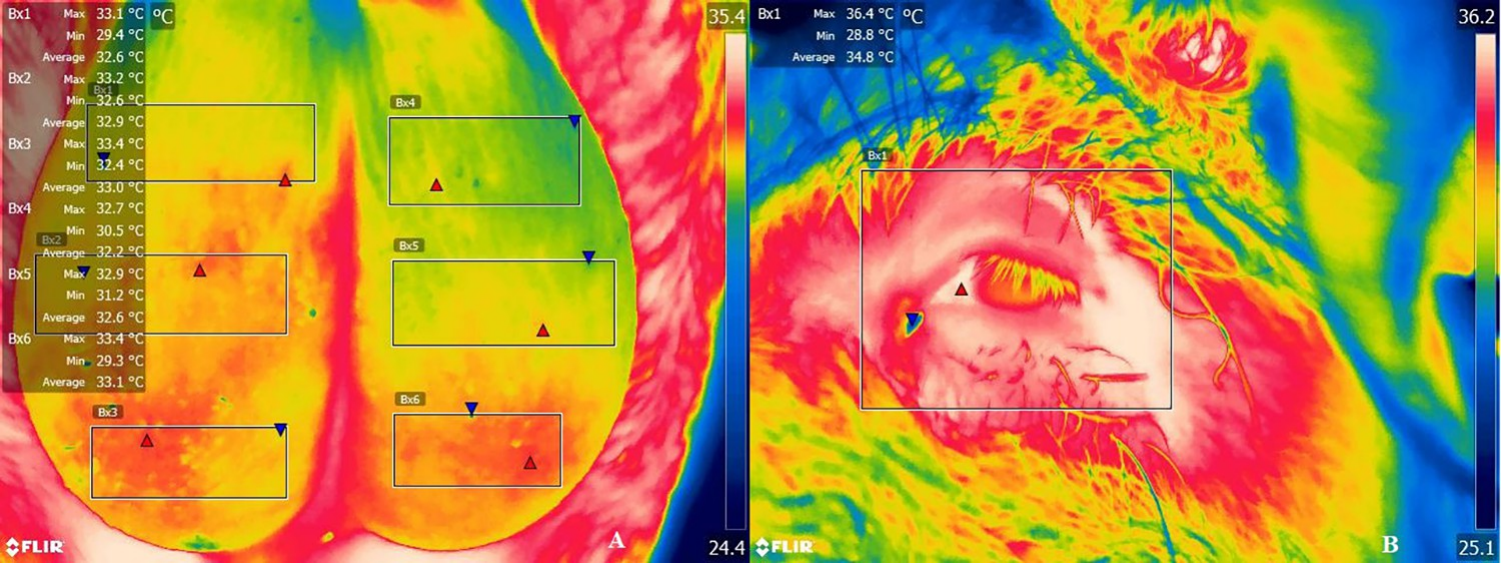
A representative thermal image from the scrotum surface (left and right testicles), with separated areas (A; top, medium, and bottom area), and from the eye (B). Red triangles indicate the highest temperature and the blue triangles indicates lowest temperature of the selected area.

The first measure allowed us to identify potential pre-existing differences prior to the treatment allocation. Afterwards, we collected thermal images weekly, on the same morning (between 9:00 and 10:15am) for all animals, one day prior to semen collection. The environmental temperature and humidity were measured with a data logger (OPUS 20 THI, 8120.00; Lufft, Germany) immediately before the thermal image was taken. We used the FLIR Quick Report Software (FLIR Systems, USA) to analyze the images, separating the dorsal, medial and ventral region of the right and left testicles (Fig 2A), and to measure the eye area temperature (Fig 2B) [17] to ensure that the animal was not in hiperthermia. We used the mean temperature taking account of every pixel from the selected area. Additionally, we evaluated the SSMT (superficial scrotal mean temperature), through a fixed central spot (FCP), given by the camera at the moment of the image collection.

### Testicular ultrasonography

The testis ultrasound scanning followed a validated methodology [13]. The evaluations of all boars were performed on the same weekday, in the morning. Initially, the boars were removed from their home pens and placed in a crate, since at least 3 minutes were necessary to complete the assessment. We performed one scanning prior to housing the animals in the experimental treatments. Subsequently, we scanned the testicles every two weeks until the end of the experiment. The left and right testicles were evaluated separately. We used the duplex B-mode (grey scale) to measure the parenchyma homogeneity, color-Doppler flow mode and spectral mode functions, using a 6 MHz convex transducer probe (Mindray, model M5Vet, Digital Diagnostic Imaging System; probe model 6LE5Vs Vet) with a topical application of a water-soluble contact gel.

To classify the B-mode ultrasound data, we used the methodology from Alves [12] and Kahwage [18]. The homogeneity of the parenchyma (HOP) was classified on a scale from 0 to 2: 0 = homogeneous parenchyma, with no pathological anechoic points; 1 = few pathological anechoic structures; 2 = heterogeneous parenchyma. With this classification, the lower score of a boar indicated a more homogeneous and healthier testicle.

Moreover, in order to analyze the vascularization of the testicular parenchyma and the pampiniform plexus, we used the same methodology from Batissaco [12]. Briefly, we used a scale for testicular parenchyma ranging from 0 to 4: 0 = no vascularization; 1 = presence of 1 or 2 vascularization points, with small-caliber vessels; 2 = presence of 3 or 4 vascularization points, with small-caliber vessels; 3 = more than 2 vascularization points, with large-caliber vessels, which was seen in 1/3 of the screen; 4 = more than 2 vascularization points, with large-caliber vessels, which was seen in 2/3 of the screen. For pampiniform plexus we used a scale from 1 to 5, which is summarized in Table 1.

**Table 1:** Score used to evaluate pampiniform plexus of boars housed in three different conditions.

Area	Score	Percentage (%)
Pampiniform plexus area filled with vascularization	1	01–20
	2	21–40
	3	41–60
	4	61–80
	5	81–100

The same person, an experienced veterinarian with a PhD in animal reproduction, performed all the measures. The ultrasound scanning aimed to verify the presence of alterations in the morphology of the parenchyma tissue, the blood flow in the parenchyma, and the blood flow in the pampiniform plexus.

### Statistical analyses

All the data were analyzed with the package Statistical Analysis System 9.4 (SAS Inst., Inc., Cary, NC). Initially, the data were checked for the presence of discrepant information (outliers) and we verified the residual normality through the Shapiro-Wilk test. After these checks, the data were analyzed by ANOVA with PROC GLIMMIX of SAS using treatment as a principal effect over the time and block (initial seminal quality) as a random effect. In addition, the command REPEATED was added to the model in order to analyze the time effect. Thus, we had the effect of treatment, the effect of time, and the interaction between these two factors.

For all analyses, 15 different covariance structures were tested and the one that best fitted the statistical model was chosen, based on the lower value of the Akaike Index correction criterion (AICC) [19]. When necessary for a post-hoc test, the Tukey’s test was used. The means and their standard error (SEM) were obtained from raw data. For all statistical analyses performed, a 5% level of significance was adopted.

## Results

The time effect without interaction with treatment was not part of our experimental design in this study and will therefore not be discussed in detail.

Analyzing the CASA data, we found a treatment effect for the variable curvilinear velocity (VCL) (see Table 2), whereby the boars housed in crates showed higher values, indicating that the sperm from these animals moved more in a circular way when compared with other treatments (p = 0.046, DF 26.5, F 4.02). For many variables, we found a time effect (progressive motility (PM), average path velocity (VAP), straight-line velocity (STR), curvilinear velocity (VCL), beat-cross frequency (BCF), and linearity (LIN)). Another important finding was regarding agglutination. We found that the boars housed in crates showed more agglutination in the semen samples comparing with boars housed in pens (p = 0.0431) and enriched pens (p = 0.0259). There was no difference between the boars housed in pens and enriched pens (p = 0.9441).

**Table 2:** The mean values and the standard error mean for the variables differently found on boars housed in three different systems (crates n = 9, pens n = 9; enriched pens n = 9).

Treatment	VCL (μm/s)	Agglutination (1–3 score)	Major defects (%)	Minor defects (%)	Total defects (%)
Crates	159.83±4.44	1.18±0.2	14.75±6.89	13.34±4.48	28.45±11.22
Pens	142.21±4.46	0.55±0.2	17.12±6.89	11.20±4.48	27.95±11.22
Enriched pens	144.53±4.71	0.46±0.21	11.42±7.04	8.29±4.57	19.35±11.56

The sperm morphology data showed no treatment effect. Many variables (abaxial tail implantation, acrosome defect, bent tail, major and minor defects, bent tail with droplet, total defects, distal droplet, and middle piece droplet) showed a time effect.

**Fig 3:**
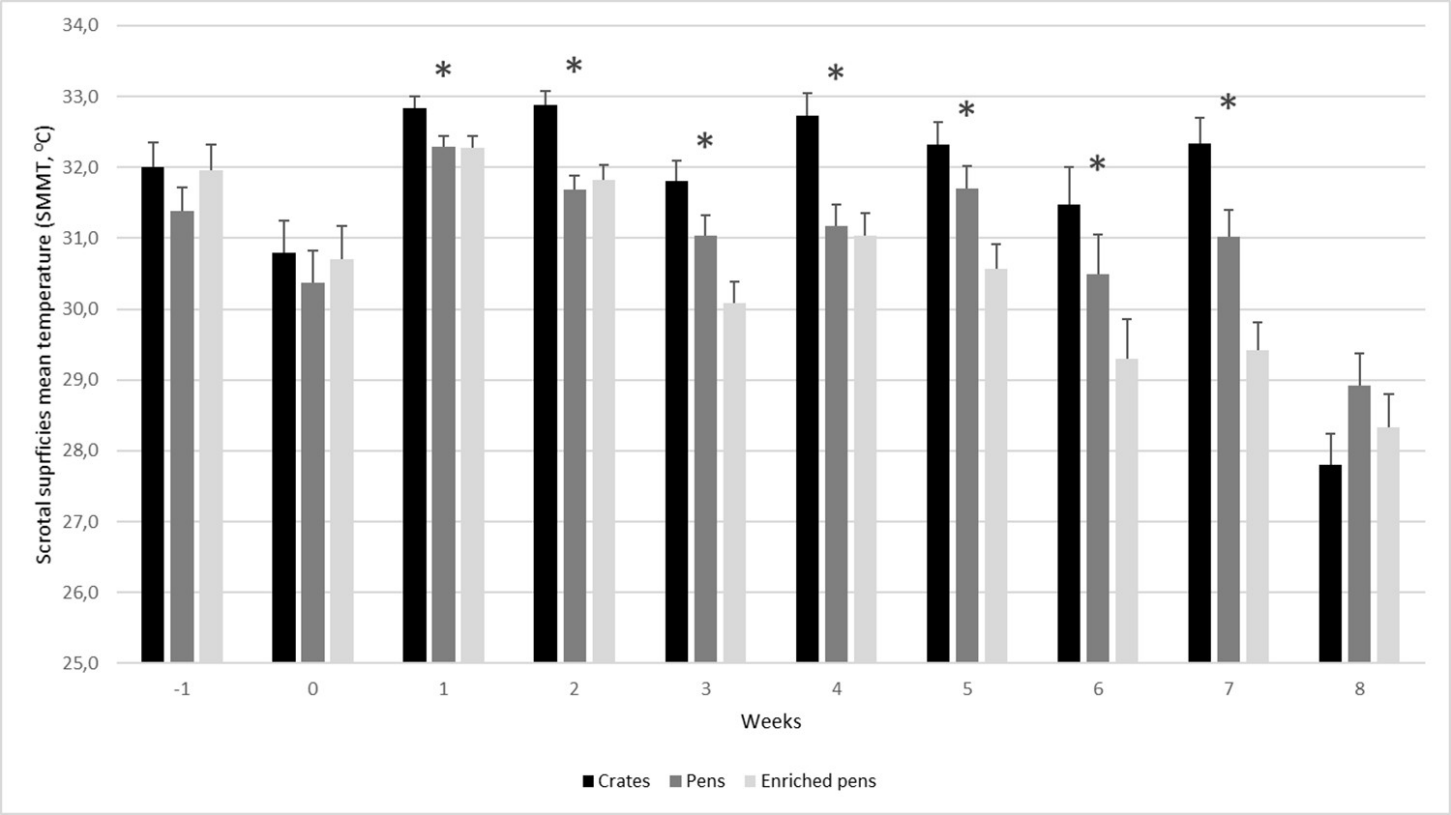
Mean environment temperature and mean humidity collected by a data logger (OPUS 20 THI, 8120.00; Lufft, Germany) at the moment of thermal pictures collection.

When considering SSMT, the animals housed in crates showed a higher temperature in the fixed central spot (FCS) than the animals housed in pens and enriched pens. No differences were observed among treatments in the week before housing them in the treatment (week -1), and in the week immediately after the treatment allocation (week 0), but there was a consistent difference in the following weeks. Additionally, the environmental temperature and the humidity was assessed at the same moment of the thermal images collection (Fig 3).

When we compared the separate regions from the testis (top, middle, and bottom area, from right and left testis), the data showed a similar pattern. For all comparisons, the animals housed in crates showed higher superficial scrotal temperature than the animals housed in pens and enriched pens. These includes the top, medium, and bottom area of the testicles and the combination (mean) value of both testicles.

The summarized data from thermal images, from the different regions averaged from both testes, are presented in the Table 3.

**Table 3:** Superficial Scrotal Mean Temperature (SSMT) and Standard Error Mean (SEM) of different areas of the scrotum from boars housed in three different conditions (Pens n = 9; crates n = 9, enriched pens n = 9).

Treatments	Mean of the superficial scrotal temperature (°C)			SEM
	Top area	Medium area	Bottom area	
Crates	31.09±0.2	31.04±0.21	31.15±0.23	0.20
Pens	30.15±0.18	30.26±0.21	30.64±0.23	0.19
Enriched pens	29.61±0.3	29.84±0.21	29.75±0.23	0.23

The data from the duplex B-mode contrast ultrasound evaluation of parenchyma homogeneity are summarized in the Table 4. The homogeneity was evaluated by a score that varies from 0 to 2.

**Table 4:** The mean score, the standard error mean, and p values of the duplex B-mode ultrasound data.

Variable	Mean score	Treatment	Time	Interaction
Left testicle	0.3431±0.1	0.1475	0.155	0.8562
Right testicle	0.3628±0.12	0.6595	0.218	0.3425
Mean	0.7059±0.2	0.2025	0.0147	0.2518

We did not identify any difference between treatment and time for the homogeneity evaluation.

The data from color Doppler ultrasound evaluation are summarized in Table 5. The tissue perfusion data were evaluated by a score that varies from 1 to 4 for parenchyma evaluation and 0 to 5 for pampiniform plexus.

**Table 5:** The mean score, the standard error mean, and p values from the color Doppler ultrasound data, which gives an indication of perfusion.

Variable		Mean score	Treatment	Time	Interaction
Left testicle	Parenchyma perfusion	2.27±0.17	<0.01	0.38	0.18
	Pampiniform plexus	2.72±0.13	0.68	0.20	0.41
Right testicle	Parenchyma perfusion	2.19±0.13	<0.01	0.18	0.01
	Pampiniform plexus	2.80±0.18	0.64	0.92	0.04
Mean parenchyma		2.23±0.15	0.01	0.09	0.01
Mean pampiniform plexus		2.75±0.22	0.90	0.51	0.14

There was no difference in the score of the pampiniform plexus. We found a difference in the parenchyma perfusion, whereby the boars housed in crates showed a higher score when compared with the boars housed in pens and enriched pens, and thus, more vessels were identified in the parenchyma of boars kept in crates. After a Tukey-Kramer test, we identified that the boars housed in crates differed significantly from boars housed in pens (p = 0.0009) and from boars housed in enriched pens (p = 0.0030). There was no difference between boars housed in pens and enriched pens (p = 0.9152).

## Discussion

The most relevant findings of the sperm analyses were the VCL and agglutination results. The VCL is relevant because it is inserted in the formula to obtain the linearity of sperm movement (LIN = VSL/VCL). Although we did not identify any difference among progressive, total, and linear motility, the VCL can compromise all these parameters. It may be that the number of animals and their age contributed to the small amount of difference among treatments. Furthermore, agglutination can compromise the motility, fertility, litter size, and, sometimes, could be a result of a tight adhesion of bacteria to the sperm cell [15, 20]. Thus, when an ejaculate is going to be used in an artificial insemination protocol, it is not recommended to use a highly agglutinated sample. The difference among treatments could therefore have an impact on boar fertility indicators.

**Fig 4:**
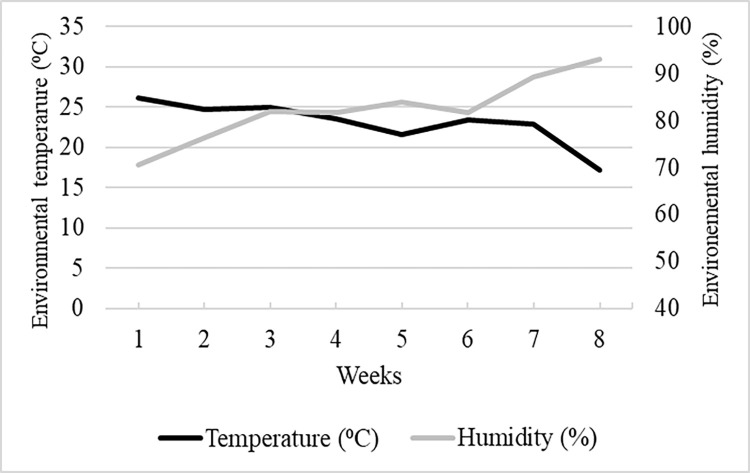
Scrotal Superficies Mean Temperature (SSMT) from boars housed in CR, PE, and EP. The data were collected weekly over the course of 10 weeks, from one week before housing until 8 weeks after. * p value <0.05.

As we presented in Fig 4, we identified that the SSMT were higher in boars housed in crates, when compared with the animals housed in pens and enriched pens. One of the possible reasons for this result is the position that the boars adopted when resting, which appears to be affected by the space available. The animals housed in crates have severe limitation in their movements which can cause abnormal behaviors [21]. Moreover, we did not observe a great variation on environmental temperature which may influence testicular temperature. Our results were consistent over the studied weeks and we attributed the changes on SSMT to the boars’ housing systems.

Previous work demonstrated that crates have a systemic effect in pigs. Sows kept in this restrictive environment showed lower quality of bone mineralization and muscle health [22]. Since they have limited movement, the posture changes may also be affected. In a previous pilot study, we observed that the boars housed in crates lie on top of their testes. Because of this, keeping in mind that these animals can reach more than 300 kg of body weight, we hypothesized that this behavior could lead to testis damage.

Our results show that the housing conditions can affect the SST, whereby the animals housed in crates showed a higher temperature. The SST is negatively correlated with sperm quality in bulls [23]. This testicular temperature elevation could lead to a pathological condition, testicular degeneration [24], which can decrease the semen quality [25]. We observed the same treatment effect when we analyzed the separate regions of the scrotum. In pigs, the epididymis cauda is in a dorsal position [26], and elevated temperature in this structure could lead to sperm modifications, important enough to alter the sperm programming [27].

The ultrasound data showed that the animals housed in crates had more blood perfusion in the parenchyma, in both testes (Table 5, mean parenchyma). This result could be an indicator that the boars had an alteration in the testis homeostasis, which increased the blood flow to that region. Note that these alterations followed the same pattern observed in the thermal images data. However, we could not observe any disturbance in the homogeneity of the parenchyma, probably because these types of lesions, observed in the ultrasonography measures, result from a chronic insult and our boars were of a young age. It might be that, if we kept them longer in the stall conditions where the insult was maintained, we would observe a more severe alteration.

## Conclusion

Restrictive housing conditions can lead to changes in superficial scrotal temperature and blood flow in the testis. Changes in temperature were observed over the scrotal area, as well as in the top, medium, and bottom sub-regions. It is important to highlight that this alteration in temperature could affect semen quality, as we reported, and influence important molecular mechanisms of stress inheritance (Bernardino et al, in preparation). Additionally, housing boars in crates can lead to an acute alteration of the blood flow to the testis parenchyma. By two weeks after the housing treatment, we were able to identify differences through color-Doppler ultrasound. We predict that, after months housed in crates, the results for blood perfusion, superficial testicular temperature, and sperm agglutination would be much more severe. In addition, boars housed in pens and enriched pens showed indicators of a better testicular health, better sperm motility features, and less agglutinated sperm than that observed in boars kept in crates. To conclude, housing boars in crates cause changes in testis physiology, which could compromise the sperm quality and the boar’s reproductive performance. On other hand, penned and enriched penned boars showed indicators of testis health and physiology.
